# Low-Temperature Crack Resistance of High-Content Rubber-Powder-Modified Asphalt Mixture under Freeze–Thaw Cycles

**DOI:** 10.3390/polym16030402

**Published:** 2024-01-31

**Authors:** Jia Guo, Chunqing Chang, Lan Wang

**Affiliations:** 1 School of Civil Engineering, Inner Mongolia University of Technology, Hohhot 010051, China; guojia990930@163.com (J.G.); ccq20090000030@imut.edu.cn (C.C.); 2 Key Laboratory of Civil Engineering Structure and Mechanics in Inner Mongolia Autonomous Region, Hohhot 010051, China

**Keywords:** asphalt mixture, low-temperature crack resistance, digital-image-processing technology, high-content rubber powder asphalt

## Abstract

In order to study the modification mechanisms of a warm-mixing agent and high dosage on rubber-powder-modified asphalt, as well as the influence of salt freeze–thaw cycling on the mechanism of warm-mixed high-dosage-rubber-powder-modified asphalt, macro- and micro-experimental methods were used to analyze the low-temperature crack resistance performance of six types of rubber-powder-modified asphalt mixtures under salt freeze–thaw cycling. By using digital image processing (DIC) technology to record and analyze the loading processes of specimens in semicircular three-point bending (SCB) tests, combined with atomic force microscopy (AFM) tests, the low-temperature crack resistance of the asphalt mixtures was explored, and it was inferred that the micro-mechanical performance indicators of the asphalt were correlated with the low-temperature crack resistance performance indicators of the asphalt mixtures. The results indicate that the salt solution caused greater damage to the asphalt than water. The addition of more rubber powder improved the low-temperature cracking resistance of the asphalt mixtures. There was a significant correlation between the micro-mechanical properties of the asphalt and the low-temperature crack resistance of the asphalt mixtures, and a dynamic mechanical thermal analyzer (DMT) showed a stronger correlation with the strain derivative ($E^{\prime }(t)$) than the adhesion force index. The SDYK-type warm-mixing agent had a better effect on the low-temperature cracking resistance of the asphalt mixtures than the EM-type warm-mixing agent.

## 1. Introduction

Due to the snow and ice on the roads in the cold regions of Northern China in the winter, the method of spreading deicing salts on road surfaces is usually used to restore traffic flow. However, this also puts road surfaces in a long-term state of freeze–thaw cycles and salt erosion, making it easier for the road structures to suffer from damage and significantly reducing their service lives under traffic loads [1]. With the rapid development of China’s automotive industry, the production of waste tires in China reached 16 million tons in 2022. The treatment and reuse of waste rubber tires have become key research topics in the rubber industry around the world [2]. Warm-mixing agents can reduce the high energy consumption and emissions caused by asphalt mixture mixing, and they can also improve the low-temperature crack resistance of asphalt pavement, extending its service life [3].

Since entering the 21st century, many scholars have conducted research on rubber-powder-modified asphalt pavement. Mena et al. found that the cost of rubber-powder-modified asphalt mixture is two-thirds that of traditional hot-mix asphalt pavement [4]. Tao Wang et al. evaluated the energy consumption and environmental impact of a warm-mixed rubber-powder-modified asphalt pavement during its lifecycle. The results indicated that the energy consumption of the warm-mixed rubber-powder-modified asphalt pavement during the maintenance phase was lower than that of a hot-mix asphalt pavement [5]. Yongchun Cheng et al. found that an asphalt mixture modified with basalt fiber diatomaceous soil had a better low-temperature cracking resistance and freeze–thaw cycling resistance, and they established a damage constitutive model for it [6]. Lövqvist Lisa et al. proposed a thermodynamic-based multi-scale model for the freeze–thaw damage of asphalt mixtures that can accurately predict and analyze damage evolution [7]. Chao Xing et al. applied DIC technology to IDT experiments and found that using digital images of smaller and the most nominal size aggregates was better for DIC deformation calculation [8]. Luo Xuedong applied DIC technology to a three-point bending test of Xiaoliang and found a strong correlation between the macro- and micro-level analysis results [9]. Zhang Yao et al. applied scanning electron microscopy technology to SCB experiments to study the fracture characteristics of fiber asphalt mixtures and to characterize their damage evolution characteristics [9]. Wang Ge proposed adding a plasticizer to activate rubber powder during the preparation of rubber-powder-modified asphalt. The performance of the rubber-powder-modified asphalt has been improved in all aspects, with the highest rubber powder content reaching 45% [10]. Wang Guoqing et al. conducted low-temperature small-beam bending tests on three different dosages (20%, 30%, and 35%) of rubber-powder-modified asphalt mortar specimens for low-temperature crack resistance testing. The study found that the low-temperature crack resistance of asphalt mortar can be effectively improved with the increase in the quality of the external rubber powder [11]. Cai Bin and others proposed the preparation method consisting of high-volume crumb-rubber-modified asphalt and analyzed its high-temperature stability, low-temperature cracking resistance, aging resistance, and other properties. The research showed that the low-temperature cracking resistance and aging resistance of high-volume crumb-rubber-modified asphalt are better than those of conventional crumb-rubber-modified asphalt, and the high-temperature stability has slightly decreased [12]. Yu Xiaoxiao et al. found that increasing the degree of degradation and regeneration of rubber powder can significantly increase its content in asphalt, and the dispersion of rubber powder particles in asphalt is more uniform, improving processing flowability and high-temperature storage stability [13]. Wang X et al. studied the adhesion performance of high-dosage rubber-powder-modified asphalt with aggregates based on the macroscopic bonding strength (BBS), the surface free energy (SFE) theory, and nano atomic force microscopy (AFM) experiments. The results showed that rubber-powder-modified asphalt had the best adhesion performance with limestone, followed by basalt, and finally, granite. Moreover, rubber powder caused the cementitious phase in asphalt to gradually decompose and decrease, reducing the adhesion of asphalt [14].

At present, there is increasing research being conducted on high-content rubber-powder-modified asphalt mixtures, but there is a lack of in-depth research on the low-temperature cracking resistance of warm-mixed high-content rubber-powder-modified asphalt mixtures. Therefore, this article focuses on the research of a warm-mixed ordinary-content rubber-powder-modified asphalt mixture, a hot-mix ordinary-content rubber-powder-modified asphalt mixture, a warm-mixed high-content rubber-powder-modified asphalt mixture, and a hot-mixed high-content rubber-powder-modified asphalt mixture. Through microscopic experiments of asphalt, the modification mechanisms of the warm-mixing agent and increasing rubber powder content on the asphalt, as well as the damage mechanism of the salt freeze–thaw cycle on the asphalt, are explored. DIC technology is applied to SCB testing, images of the loading process of the specimen are collected, and the fracture characteristics of the rubber-powder-modified asphalt mixtures are analyzed under different salt freeze–thaw cycles from both macro- and micro-perspectives. This study can provide a reference for the use of asphalt pavement materials in cold northern regions and areas with large temperature differences.

## 2. Materials and Methods

### 2.1. Raw Materials

The testing materials and test markings in this article are listed in Table 1.

Panjin 90 # base asphalt was selected for the experiment. The modifier was 60-mesh rubber powder particles, with the rubber powder content accounting for 20% and 30% of the mass of the matrix asphalt. The performance indicators of the rubber powder are shown in Table 2. For the warm-mixing agent, we used the SDYK surface active dosage form and the EM viscosity-reducing dosage form developed by the Shandong Institute of Transportation Science. And the warm-mixing agent adopted the external mixing method with dosages of 0.6% and 1% of the mass of rubber-powder-modified asphalt. The preparation process of warm-mixed 20% rubber-powder-modified asphalt (WCR-20, including ECR-20 and SCR-20) is as follows: The first step is to heat the base asphalt in an oven until it has good fluidity. Then, the base asphalt is quickly placed on a heating and stirring instrument, with the instrument temperature controlled at around 140 °C. Rubber powder particles are added step by step, and after all the rubber powder is added, the stirring temperature is strictly controlled at 180 °C to 190 °C with continued stirring for 30 min. The second step is to place the mixture on a high-speed shearing machine after stirring for 20 min, with a shearing speed of 7000 r/min. The heating temperature during the shearing process is strictly controlled at 160 °C. After shearing, it is placed in an oven for 1 h to develop and produce CR-20. The third step is to place the modified asphalt with rubber powder on a mixing instrument for mixing. At this time, the mixing temperature should be controlled at around 165 °C. During the mixing process, SDYK or EM warm-mixing agent should be gradually added. After all of the warm-mixing agent is added, continue stirring for 5 min to fully integrate it into the modified asphalt with rubber powder, and prepare SCR-20 and ECR-20, respectively. The preparation process of warm-mixed high-content rubber-powder-modified asphalt (WCR-30, including ECR-30 and SCR-30) is similar to that of WCR-20. The difference is that the first step is to add activated 15% rubber powder to the base asphalt for stirring. After stirring for 30 min, 15% rubber powder and 2% SBS are added to the asphalt and stirred for another 30 min. The temperature during the stirring process is strictly controlled between 180 °C and 190 °C. After mixing, the asphalt is subjected to high-speed shearing, which is the same as that of 20% rubber-powder-modified asphalt. The difference is that during the shearing process of the high-content rubber-powder-modified asphalt, the temperature is controlled between 180 °C and 190 °C. After shearing, 0.2% stabilizer (sulfur) is added to the asphalt, and the asphalt is placed in an oven for 3 h to develop into CR-30. The process of the third step is the same as that of WCR-20, and the final products are ECR-30 and SCR-30. For the rubber-powder-modified asphalt mixture, we applied AC-16 grading. Coarse and fine aggregates were obtained from basalt, and the mineral powder was limestone mineral powder. The deicing salt used in the salt freeze–thaw cycle test was industrial salt (NaCl).

The technical indicators of warm-mixed rubber-powder-modified asphalt and warm-mixed high-content rubber-powder-modified asphalt are shown in Table 3 and Table 4.

### 2.2. Mix Design

Both coarse and fine aggregates were selected as basalt, which was divided into three grades: 0–5, 5–10, and 10–20. The mineral powder was limestone, and the grading was AC-16 type. The volume index under optimal asphalt dosage and mix design results are shown in Table 5 and Table 6.

### 2.3. Preparation of Test Pieces

**Figure 1:**
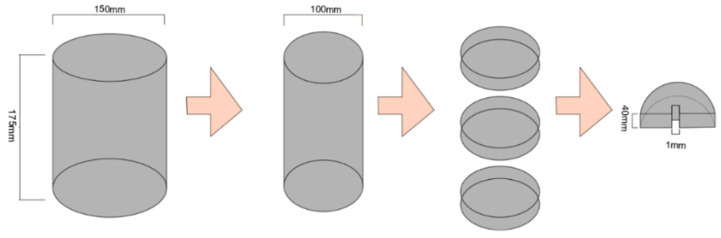
Semicircular specimen preparation steps.

According to the research results of the research conducted in [9], the mixing and compaction temperatures of the warm-mixed asphalt mixture were determined using the equal void ratio method, followed by a variable-temperature Marshall test. Cylindrical specimens with a diameter of 150 mm and a height of 175 mm were formed using the gyratory compaction method. Then, core samples were taken from the cylindrical specimens to obtain cylindrical specimens with a diameter of 100 mm and a height of 175 mm. Three cylindrical slices with a thickness of 40 mm were cut from the middle of each specimen, and each slice was cut into two semicircular shapes with the same radius. In order to better utilize DIC technology to capture and analyze the cracking process of the specimens, a 5 mm deep and 1 mm wide pre-cut notch was made at the center of the bottom of each semicircular cut edge, as shown in Figure 1.

### 2.4. Design of Freeze–Thaw Cycle Test

#### 2.4.1. Design of Asphalt Freeze–Thaw Cycle Test

Based on the relevant results of the research conducted in [15], as well as the freeze–thaw cycle test of the asphalt mixtures in this paper, combined with the rapid freezing method of cement concrete, asphalt water freeze–thaw cycle and salt freeze–thaw cycle tests were conducted. First, 50 g ± 0.5 g of the asphalt sample was weighed and placed in a stainless steel container, with the thickness of the asphalt film controlled at 3.2 mm ± 0.2 mm. After the asphalt was cooled, pure water and an 8% concentration salt solution were injected until the asphalt solidified, and the container was sealed with a film. Finally, the container containing the asphalt was placed in a high- and low-temperature alternating test chamber and frozen for 2 h at −20 °C. Then, the temperature was increased to 60 °C ± 0.5 °C and maintained for 4 h, completing a freeze–thaw cycle. Due to the fact that asphalt testing was conducted to allow for an analysis of the test results to effectively validate the asphalt mixtures, only 20 freeze–thaw cycles were conducted, with salt solution concentrations of 0% and 8%.

#### 2.4.2. Design of Freeze–Thaw Cycle Test for Asphalt Mixture

This article considers the characteristics of extreme weather in the Inner Mongolia region, as well as relevant research on the freeze–thaw cycles of asphalt mixtures [16,17]. The process of the water freeze–thaw cycle and salt freeze–thaw cycle tests for the asphalt mixtures is as follows: First, place the prepared semicircular test piece in pure water and 8% concentration salt solution for 15 min under vacuum saturation (vacuum degree of 97.3–98.7 kPa), restore normal pressure, and allow to stand for 1 h. Then, place the test piece in a test box and inject pure water and an 8% concentration salt solution until there is no test piece left. Finally, seal the test box with a film to preserve the water, and freeze it in a high- and low-temperature alternating test chamber at −20 °C for 8 h. After the freezing is completed, raise the temperature to 60 °C ± 0.5 °C and hold it for 16 h, completing a freeze–thaw cycle. The freeze–thaw cycle was repeated for 5, 10, 15, and 20 cycles, with two salt solution concentrations (0% and 8%) used for comparison.

### 2.5. SCB Test and Digital Image Processing Technology

The semicircular bending tensile test was first proposed in 1984, and Chong et al. used it to determine the fracture toughness of rocks. In recent years, it has gradually been widely used by researchers to evaluate the performance of asphalt pavement materials [9,17]. The SCB test uses a UTM-100 testing machine to load six types of semicircular asphalt mixture specimens. The control mode is strain control mode, with a loading rate of 0.75 mm/min. The test is stopped when the load reaches 10% of the peak load. The crack propagation type is an open type, with a radius of 50 mm and a thickness of 40 mm. The experimental temperature is −10 °C. Due to the synchronous nature of SCB testing and DIC data collection, it is necessary to consider the impact of the loading rate on DIC data collection. A too fast loading rate can cause the specimen to fracture too quickly, affecting DIC data collection and observation. A slow loading rate can lead to a large amount of DIC data and difficulties in data processing. After extensive preliminary experiments, it has been determined that a loading rate of 0.75 mm/min can ensure stable crack propagation and facilitate DIC data collection and processing. The purpose of stopping the experiment when the load reaches 10% of the peak load is to obtain a more complete load displacement curve of the asphalt mixture, which is convenient for the subsequent calculation of quantitative indicators of asphalt mixture crack resistance performance.

**Figure 2:**
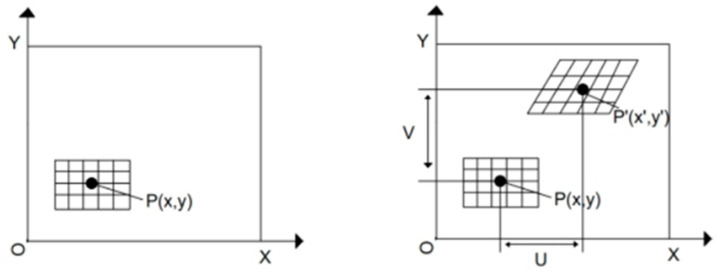
Speckle diagram before and after deformation.

DIC is based on the principle of physical optical interference. After light is diffusely reflected on the surface of an object, it forms a random particle structure in spatial interference [18], as shown in Figure 2. Before deformation, the speckle subregion on the image is first identified, with the center point of the speckle reference subregion being P (x, y). A correlation calculation is performed on the subregions of the image before and after deformation, and the center point P (x′, y′) of the target subregion is calculated using the correlation function. Finally, based on the calculation results, the full-field displacement can be obtained, and the Cauchy equation can be applied to transform the full-field displacement into the full-field strain.

### 2.6. Atomic Force Microscopy

The micro-mechanical properties of the original, aged, and recycled asphalt were observed and tested at room temperature using atomic force microscopy (AFM) with a scanning area of 15 μm×15 μm. The pixel size was 512×512, and the QNM scanning mode was used, which is a patented technology launched by Bruker Company that can obtain Young’s modulus (DMT) and adhesion. Adhesion can be used to evaluate the adhesion performance of the asphalt–aggregate interface, providing a reference for a macroscopic evaluation of the water damage resistance of the asphalt mixtures. Due to the fact that silicon dioxide (${SiO}_{2}$) is the main component of aggregates, silicon probes were used to simulate the characteristics of the interfacial adhesion between the mineral aggregates and asphalt.

## 3. Results and Discussion

### 3.1. Macroscopic Analysis of Crack Resistance Performance

**Figure 3:**
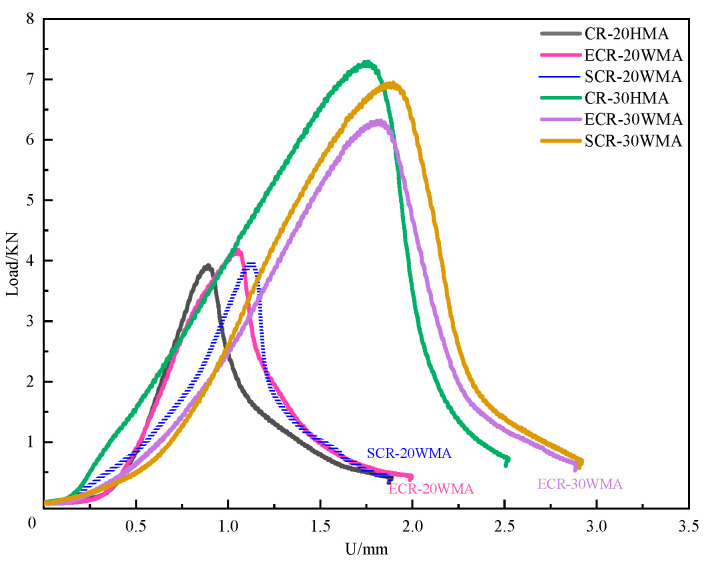
Load–displacement curves of 6 types of crumb-rubber-modified asphalt mixture under SCB test.

Figure 3 shows the load–displacement curves of six types of rubber-powder-modified asphalt mixtures. In Figure 3, it can be seen that the load–displacement curves of the rubber-powder-modified asphalt mixtures can be divided into two stages based on the peak load. In the first stage, the displacement increases with the increase in applied stress. As the accumulated stress on the asphalt mixture exceeds its ultimate tensile strength, the load reaches its peak and enters the second stage. In this stage, the specimen cracks, and the fracture resistance of the asphalt mixture rapidly decreases, causing the applied stress to decrease with the increase in displacement. The first stage of SCR-30WMA is characterized by a forward displacement smaller than that of the other five types of rubber-powder-modified asphalt mixtures. The curve slope is also more gentle, and the duration of the entire fracture process is longer. Therefore, the deformation ability and stress relaxation ability of the rubber-powder-modified asphalt mixture improve [19].

**Figure 4:**
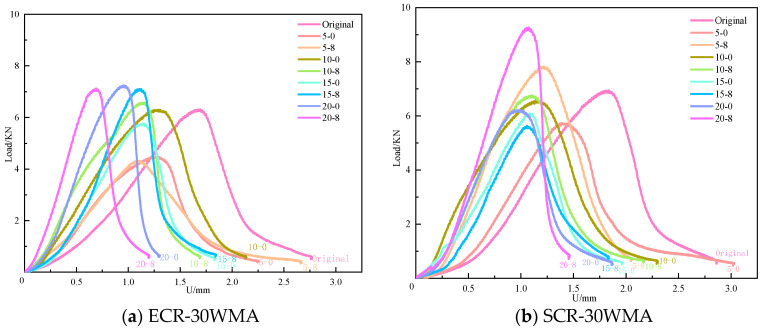
Modification of warm-mixed high-content rubber powder under freeze–thaw cycle load–displacement curve of asphalt mixture.

Figure 4 shows the modification of warm-mixed high-content rubber powder under the freeze–thaw cycle load–displacement curve of the asphalt mixture. As shown in Figure 4, when using the same salt solution concentration, the magnitude of the forward shift of the first-stage curve of SCR-30WMA increases with the increase in freeze–thaw cycles. This indicates that the viscosity of the asphalt mixture begins to transition from elasticity after the freeze–thaw cycles, leading to a decrease in its deformation ability and an earlier occurrence of fracture. This is because, as the number of freeze–thaw cycles increases, an increasing number of water molecules enter the interior of the asphalt film. The water aging caused by the water molecules on the asphalt results in a decrease in the adhesion between the asphalt and the aggregates. When the temperature drops from high to low again, the water molecules entering the interior of the mixture form ice crystals, causing the volume of the mixture to expand. The freezing and expansion effect of the water leads to a loose structure of the asphalt mixture skeleton and a decrease in the interaction force between the asphalt-and-aggregate interface, making the specimen more prone to failure. Therefore, the low-temperature fracture resistance of the rubber-powder-modified asphalt mixture decreases with the increase in freeze–thaw cycles.

In Figure 4, it can also be seen that, under the same number of freeze–thaw cycles, the first-stage curve of the specimens subjected to salt freeze–thaw cycles moves forward more significantly than that of the specimens subjected to water freeze–thaw cycles. This is because salt ions have a stronger polarity than asphalt, and salt ions have a stronger adsorption capacity for aggregates than asphalt. At this point, the peeling effect of ${Na}^{+}$ and ${Cl}^{-}$ in the solution on asphalt reduces the adhesion of the asphalt to the aggregates [20], leading to a decrease in the deformation ability of the rubber-powder-modified asphalt mixture. The fracture characteristics of the material are similar to those of elastic materials in low-temperature environments and under external loads.

### 3.2. Microscopic Analysis of Crack Resistance Performance

**Figure 5:**
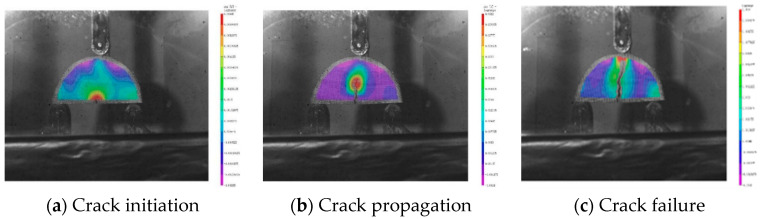
SCR-30 WMA horizontal strain characteristic cloud chart.

Figure 5 shows the changes in the horizontal strain field (Exx) cloud map of the crack in the initiation, propagation, and failure stages during the loading process of the SCR-30WMA semicircular specimen; this map was collected using DIC equipment and processed and calculated using VIC-3D 9 software. In Figure 5, the closer the red area, the stronger the horizontal tensile stress and the greater the horizontal tensile deformation of the specimen; the closer the purple area, the stronger the horizontal compressive stress and the greater the horizontal compressive deformation of the specimen. 

In Figure 5a, it can be seen that during the crack initiation stage, the EXX values of the specimen increased from top to bottom. In this stage, the specimen was under compression at the top and under tension at the bottom. A localized horizontal strain concentration zone (indicated by the red area in the figure) appeared at the pre-cut notch, and a crack tip formed at the top of the specimen, indicating the initiation of a microcrack. In Figure 5b, in addition to the horizontal strain concentration zone, it can be observed that, during the crack propagation stage, as the load continued to increase, the specimen was under compression overall. The horizontal strain concentration zone moved upward as the macroscopic crack extended. In Figure 5c, it can be seen that during the failure stage, the localized horizontal strain concentration zone continued to move along the crack until the crack penetrated the specimen, and the horizontal strain concentration zone disappeared at the top of the specimen.

**Figure 6:**
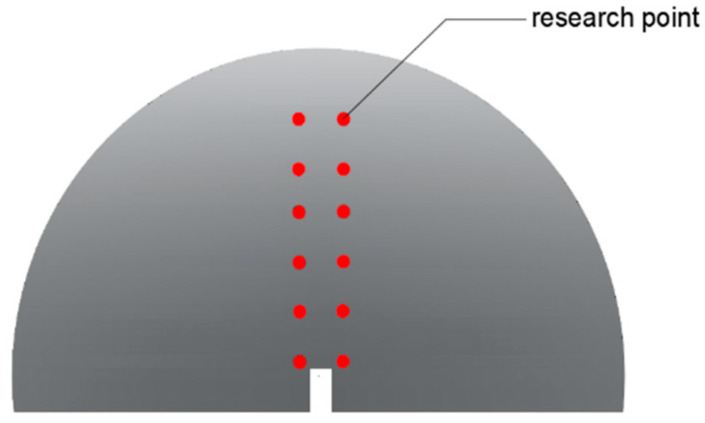
Research point selection diagram.

Based on the analysis of the horizontal strain characteristics of the rubber-powder-modified asphalt mixture, it was determined that the localized horizontal strain concentrated area was mainly in the form of a circle with a radius of 5 mm centered at the crack tip. In order to avoid missing data points too close to the crack position and data points deviating from the strain concentration area when they are too far away, two symmetric columns of data points located 2 mm away from both sides of the crack initiation point were selected as the research points for the cracking area (as shown in Figure 6). Since Type I cracks usually occur under horizontal tensile stress, compared with the vertical strain–time (Eyy-t) curve, the Exx-t curve can better reflect the cracking characteristics of the rubber-modified asphalt mixture [21]. Therefore, in this study, the Exx-t curve was used to analyze the cracking characteristics of the asphalt mixture.

**Figure 7:**
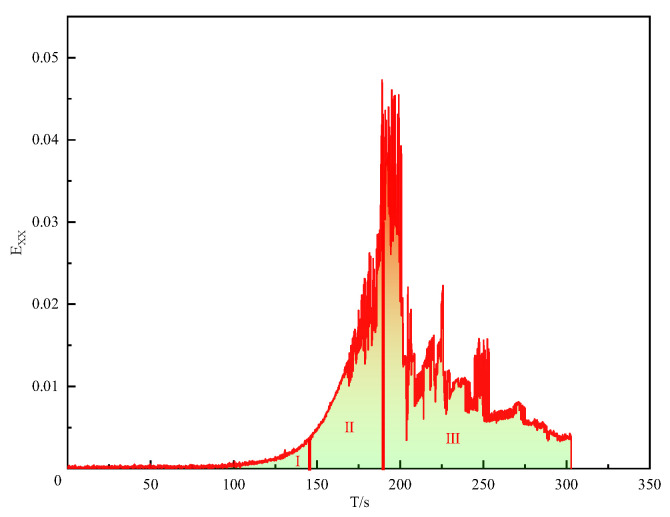
Exx-t curve of SCR-30WMA.

As shown in Figure 7, the Exx-t curve of SCR-30WMA divides the cracking process into three stages: The first stage is the stable growth stage of the strain, where the specimen could resist the bending and tensile stress generated by the load, and only a small amount of deformation occurred without crack formation. The second stage is the rapid growth stage of the strain, where, as the load continued to increase, the specimen reached its ultimate bending and tensile strength, and microcracks were generated at the top of the pre-cut notch and continuously developed into macroscopic cracks, with a horizontal strain concentration zone appearing at the crack initiation point. The third stage is the decrease in the strain, where, as the specimen completely released the stress caused by cracking, the horizontal strain decreased with the release of stress, the macroscopic crack rapidly expanded upward until the specimen was completely destroyed, and the localized horizontal strain concentration position moved upward until it disappeared.

**Figure 8:**
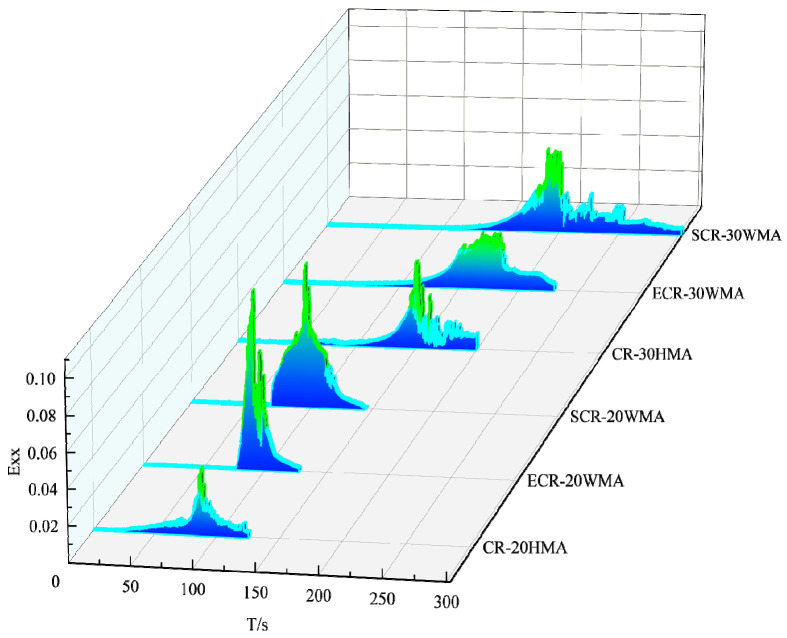
Exx-t curves of the original 6 asphalt mixes.

Based on the occurrence time of the second stage of Exx-t, which is the moment when the specimen undergoes cracking behavior, the influence of the amount of warm-mixing agent and rubber powder on the cracking characteristics of the asphalt mixtures is analyzed. Figure 8 shows the Exx-t curves of the six types of asphalt mixtures.

In Figure 8, it was observed that ECR-20WMA appeared later than CR-20HMA in the second stage, and ECR-30WMA also appeared later than CR-30HMA in the second stage, indicating a slower crack propagation rate. The reason for this was that the addition of EM in the asphalt mixture effectively improved the stress relaxation ability of the mixture, enhancing its flexibility and reducing the stress concentration, thus delaying the occurrence and development of cracks and improving the crack resistance of the asphalt mixture. It was also observed that SCR-20WMA appeared later than CR-20HMA in the second stage, and SCR-30WMA appeared later than CR-30HMA in the second stage, indicating that the addition of SDYK improved the low-temperature crack resistance of the asphalt mixture. This was because SDYK, as a surfactant, had polarity and charge, and the aggregate surface also had charge under normal conditions. Therefore, with the increase in the mixing time between the asphalt and the aggregate [21], the attraction between the positive and negative charges allowed for the asphalt to better coat the aggregate surface, enhancing the continuity of the internal structure of the asphalt mixture and improving its deformation ability. As a result, external stress could be distributed more uniformly throughout the asphalt mixture, reducing the impact of the stress concentration and effectively slowing down the crack propagation rate. It was observed that ECR-20WMA appeared earlier than SCR-20WMA in the second stage, and ECR-30WMA appeared earlier than SCR-30WMA in the second stage, indicating that the addition of SDYK had a better effect on the crack resistance of the asphalt mixture than EM. This was because EM exists in a crystalline form in low-temperature environments, and this causes changes in the asphalt structure and a decrease in the adhesion between the asphalt and the aggregates, making the asphalt mixture more prone to cracking under external stress.

CR-30HMA appeared later than CR-20HMA in the second stage, and ECR-30WMA also appeared later than ECR-20WMA in the second stage. Additionally, SCR-30WMA appeared later than SCR-20WMA in the second stage. Therefore, increasing the dosage of rubber powder can effectively improve the crack resistance of asphalt mixtures. This is because the increased dosage of the rubber powder forms a larger and more complex network structure after swelling in asphalt. The interaction forces between the network structures in the asphalt are stronger, resulting in greater surface roughness. This enhances the adhesion between the asphalt and aggregates, effectively slowing down the crack propagation speed. Furthermore, rubber powder has good elasticity, and when the dosage is high, it can effectively reduce the stress concentration in asphalt mixtures, thereby improving the crack resistance performance of asphalt mixtures.

The strain derivative index $E^{\prime }(t)$ is used for evaluation, where $E^{\prime }(t)$ is the ratio of *E_YY_* to *E_XX_* per unit time, which characterizes the relative deformation of the asphalt mixture per unit time under the three-point bending tensile loading mode. The smaller the value of $E^{\prime }(t)$, the closer it is to the cohesive body in low-temperature environments, and the better its crack resistance performance. The function expression is outlined in (1) [22]:

(1) $$E^{\prime }\left(t\right)=\frac{E_{YY}(t)}{E_{XX}(t)}\times \frac{1}{t}$$

In the equation, $E_{XX}(t)$ is the horizontal strain–time curve function; $E_{YY}(t)$ is the vertical strain–time curve function; and *t* is the time.

When Exx reaches its maximum, that is, when macroscopic cracks form in the asphalt mixture, the specimen undergoes fracture failure, which is more suitable for analyzing the crack resistance performance of the asphalt mixture. Therefore, *t* in Formula (1) is the time when Exx reaches its maximum value.

**Figure 9:**
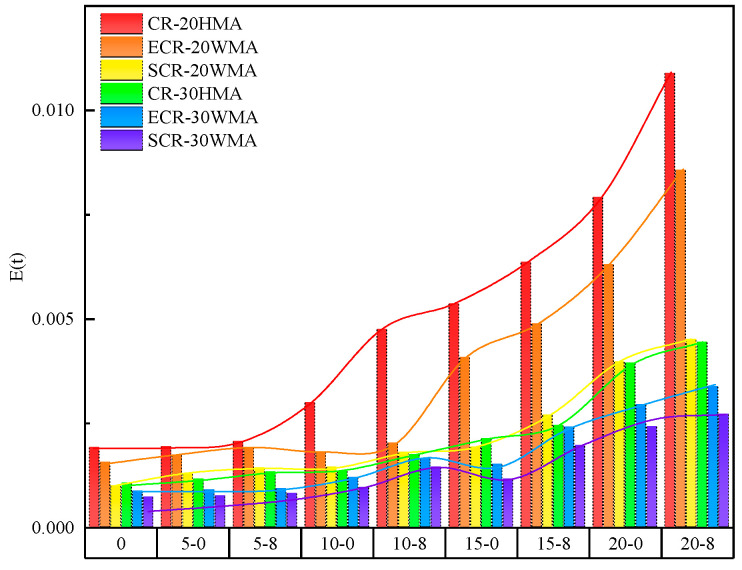
$E^{\prime }\left(t\right)$ of rubber-powder-modified asphalt mixture under salt freeze–thaw cycles.

Figure 9 shows the values of the different rubber-powder-modified asphalt mixtures under various freeze–thaw cycles. According to the analysis in Figure 9, under the same freeze–thaw cycle, the order of values from largest to smallest is as follows: CR-20HMA > ECR-20WMA > SCR-20WMA > CR-30HMA > ECR-30WMA > SCR-30WMA. This indicates that the high-dosage rubber-powder-modified asphalt mixtures had a better fracture resistance in low-temperature environments. The essence of this phenomenon is twofold. On the one hand, increasing the dosage of the rubber powder enhanced the adhesive properties of the rubber-powder-modified asphalt, making the mixture more resistant to cracking under external forces. On the other hand, the addition of the warm-mixing additives not only improved the adhesion between the asphalt and aggregates but also enhanced the deformability of the rubber-powder-modified asphalt mixture. Among them, SCR-30WMA had better fracture resistance than ECR-30WMA, mainly due to the different interaction mechanisms with the asphalt. The polar groups in the SDYK surfactant were easily adsorbed by the asphalt resin, resulting in a relatively loose asphalt aggregate structure, which exhibited certain fluidity at low temperatures [23] and had a small impact on the viscosity of the asphalt. The EM viscosity reducer formed a new aggregate structure through a molecular interaction with the asphalt, creating a stable spatial structure that made the asphalt less prone to deformation. This led to an increase in the dispersion of the asphalt molecular structure, thereby reducing the viscosity of the asphalt [24].

According to Figure 9, the values of the various rubber-powder-modified asphalt mixtures increased with the increase in freeze–thaw cycles with the same salt solution concentration. This indicates that, during the loading process of the asphalt mixtures in low-temperature environments, the freeze–thaw cycles accelerated the transition of the material from viscous to elastic, making the specimens more prone to damage. At the same time, under the same freeze–thaw cycles, the values of the specimens after the salt freeze–thaw cycle were larger than those after the water freeze–thaw cycle, indicating that the coupling effect of freeze–thaw cycles and salt erosion made the fracture characteristics of the rubber-powder-modified asphalt mixtures more similar to those of linear elastic materials, resulting in a significant decrease in the fracture resistance.

### 3.3. Microscopic Properties under AFM

**Figure 10:**
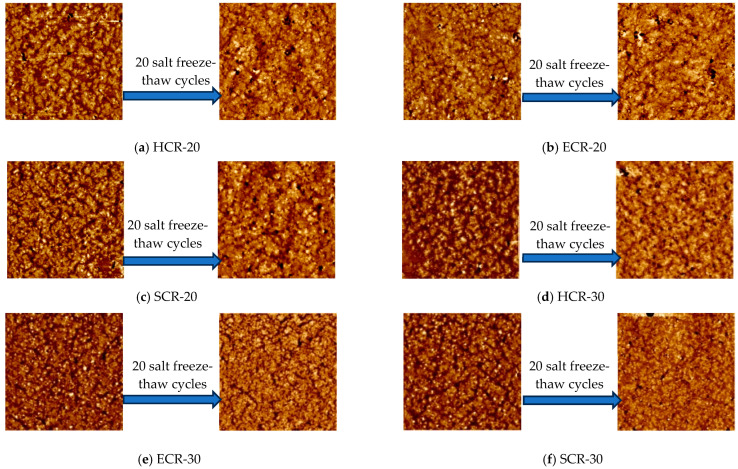
Microscopic morphology of crumb-rubber-modified asphalt before and after 20 salt freeze–thaw cycles.

Figure 10 shows the morphological images of the six types of rubber-powder-modified asphalt before and after the salt freeze–thaw cycles. In Figure 10, it can be observed that all six types of rubber-powder-modified asphalt did not exhibit an obvious “honeycomb structure”, but instead showed a chain-like structure with three colors: black, brown, and white. This was because, after the addition of the rubber powder to the asphalt, the light components and some wax in the asphalt were absorbed by the rubber powder, resulting in a reduction in the wax content that formed the “honeycomb structure” [25]. The asphalt surface was scattered with larger black particles and white bright spots, which was caused by the asphalt adhering to the surface of the rubber powder and the temperature being insufficient to achieve a molten state between the rubber powder and the asphalt, resulting in agglomeration. However, increasing the preparation temperature would lead to the severe aging of the asphalt, a loss of oil content, and the generation of smoke that is harmful to the environment and human health.

The analysis in Figure 10 revealed that the high-dosage rubber-powder-modified asphalt had a higher number of chain-like structures and a more uniform distribution of white bright spots (rubber powder particles) than the normal-dosage rubber-powder-modified asphalt. This was because, with a higher dosage of rubber powder, the high-molecular-weight polymers in the rubber powder are more densely distributed in the asphalt, forming more cross-linking and aggregation points, resulting in a more stable network structure. Compared to the normal-dosage rubber-powder-modified asphalt, the high-dosage rubber-powder-modified asphalt had a more complex network structure with tighter cross-linking, thus exhibiting a better bonding strength. Additionally, the high-dosage rubber-powder-modified asphalt had a higher deformation capacity, as the cross-linking between the rubber powder molecules could absorb more deformation energy, thereby enhancing the low-temperature crack resistance of the asphalt.

The chain-like structure of the modified asphalt with six types of rubber powder significantly decreased after 20 freeze–thaw cycles, and the white bright spots were greatly diminished, replaced by more black granular particles. The reason behind this is that the rubber powder particles tended to agglomerate after the freeze–thaw cycles, making it difficult to observe individual rubber powder particles and their chain-like structures in the morphology image. The dispersed medium also became rougher, resulting in a reduced uniformity in the rubber powder asphalt and water aging phenomenon. The connection between the rubber powder particles and the asphalt matrix weakened, leading to a decreased flexibility in the asphalt at low temperatures [26].

**Figure 11:**
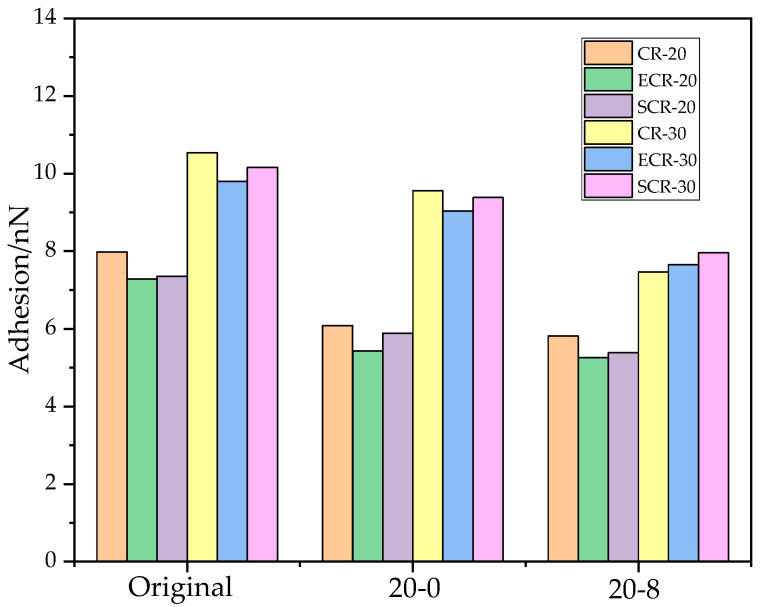
Adhesion of asphalt under freeze–thaw cycle.

In Figure 11, it can be seen that the adhesion of ECR-20 was lower than that of CR-20, and the adhesion of ECR-30 was lower than that of CR-30, indicating that the addition of EM reduced the adhesion of the asphalt to the aggregates. This is because EM reduced the intermolecular forces of the asphalt, decreased the surface roughness, and reduced the adhesive area. The adhesion of SCR-20 was lower than that of CR-20, and the adhesion of SCR-30 was lower than that of CR-30. This is because the addition of SDYK to the asphalt resulted in the formation of a charged water film on the surface, which lubricated the asphalt structure and reduced the surface roughness, resulting in a decrease in the adhesive area. Therefore, the addition of SDYK also reduced the adhesion of the asphalt to the aggregates. The adhesion of ECR-20 was lower than that of SCR-20, and the adhesion of ECR-30 was lower than that of SCR-30. This is because the addition of EM increased the light components of the asphalt, reduced the surface roughness, and decreased the adhesion. However, the addition of SDYK to the asphalt did not significantly increase its light components, but instead, lubricated its surface. Moreover, the addition of EM reduced the surface roughness of the asphalt more than the addition of SDYK. Therefore, the impact of EM on the adhesion of asphalt is greater than that of SDYK.

The adhesion of CR-30 was greater than that of CR-20, ECR-30 had greater adhesion than ECR-20, and SCR-30 had greater adhesion than SCR-20, indicating that increasing the dosage of the rubber powder resulted in an increase in the adhesion of the asphalt. The reason for this was that increasing the dosage of the rubber powder made the surface of the asphalt rougher, thereby increasing the adhesive area and improving the adhesion with the aggregate, thus enhancing the crack resistance of the asphalt mixture. 

The adhesion forces of the six types of asphalt were reduced after the freeze–thaw and salt freeze–thaw cycles. This was because the freeze–thaw cycles intensified the erosion of the water molecules and salt ions on the asphalt, causing changes in the apparent structure of the asphalt and a decrease in the surface roughness, resulting in a reduction in the adhesive area. Additionally, the salt freeze–thaw cycles further decreased the surface roughness of the asphalt, leading to a decrease in the adhesion forces. 

**Figure 12:**
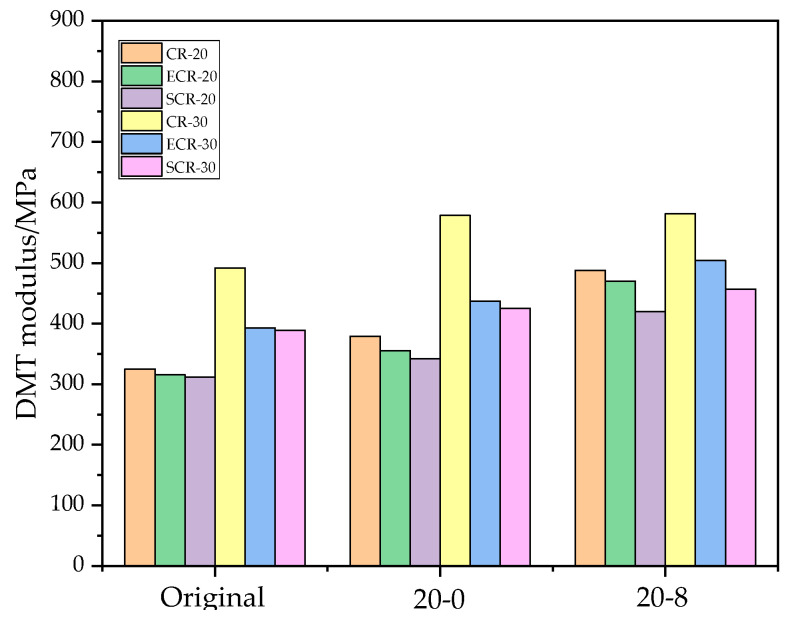
DMT modulus of asphalt under freeze–thaw cycle.

In Figure 12, it can be observed that the DMT modulus of ECR-20 was lower than that of CR-20, and the DMT modulus of ECR-30 was lower than that of CR-30, indicating that the addition of EM improved the deformation ability of the asphalt. This is because the addition of EM increased the light components in the asphalt, enhanced its flowability, and provided a better deformation ability at low temperatures. Furthermore, Table 3 and Table 4 show that the elongation of ECR-20 and ECR-30 was higher than that of CR-20 and CR-30. The DMT modulus of SCR-20 was lower than that of CR-20, and the DMT modulus of SCR-30 was lower than that of CR-30. This is because, when SDYK was added, it acted as a lubricant in the asphalt structure, reducing the surface roughness, enhancing flowability, and providing a better deformation ability at low temperatures. It also improved the uniform adhesion of the asphalt to the aggregates, thereby enhancing the crack resistance of the asphalt mixtures. The DMT modulus of ECR-20 was higher than that of SCR-20, and the DMT modulus of ECR-30 was higher than that of SCR-30, indicating that the addition of SDYK to the asphalt improved its deformation ability more than the addition of EM. This is because at normal or low temperatures, EM exists in the form of crystals in asphalt, causing some changes in the asphalt structure and reducing its flexibility. Therefore, the addition of warm-mixing additives can improve the low-temperature crack resistance of asphalt mixtures, and SDYK has a better effect on the low-temperature crack resistance of asphalt mixtures than EM.

The DMT modulus of CR-30 was greater than that of CR-20, and the DMT modulus of ECR-30 was greater than that of ECR-20. Additionally, the DMT modulus of SCR-30 was greater than that of SCR-20. This indicates that the incorporation of more rubber powder resulted in a decrease in the deformability of the asphalt. This was because increasing the amount of rubber powder led to a denser internal structure and a rougher surface of the asphalt, reducing the free space. Therefore, CR-30 was harder and had slightly poorer deformability.

The DMT modulus of all six types of asphalt increased after the freeze–thaw cycles and salt freeze–thaw cycles. This was due to the intensified erosion of the water molecules and salt ions on the asphalt during the freeze–thaw cycles, causing the asphalt to become harder and reducing its flowability and deformability. The increase in the DMT modulus after the salt freeze–thaw cycles was greater than that after the water freeze–thaw cycles, indicating that, under the same number of freeze–thaw cycles, the damage caused by the salt solution to the asphalt was greater than that caused by water. This made the asphalt harder and reduced its deformability, thereby negatively affecting the low-temperature crack resistance of the asphalt mixture.

## 4. Correlation Analysis

The principle of the grey correlation analysis was based on inferring the degree of correlation between variables in a system based on the changes in the observed values [27]. Therefore, in this study, the strain derivative $E^{\prime }(t)$ at a micro-scale was selected as the reference sequence, while the flexibility index, FI, and crack resistance index, CRI, at a macro-scale were chosen as the comparative sequences. The correlation between them was analyzed using a grey correlation analysis. The specific steps of the grey correlation evaluation system were as follows:

(1) Determine the reference sequence and comparison sequence:
  (2) $$\mathrm{The}\;\mathrm{reference}\;\mathrm{sequence}\;\mathrm{is}\;X_{i},X_{i}=\left[x_{i}\left(1\right),x_{i}\left(2\right),\ldots ,x_{i}(n)\right]$$
  (3) $$\mathrm{The}\;\mathrm{comparison}\;\mathrm{sequence}\;\mathrm{is}\;X_{j},X_{j}=\left[x_{j}\left(1\right),x_{j}\left(2\right),\ldots ,x_{j}(n)\right]$$
(2) Perform the dimensionless processing of the reference sequence and comparative sequence.
  The original data need to be dimensionally eliminated, i.e., normalized and converted into a comparable sequence of numbers.
  The dimensionless reference sequence is
  (4) $$Y_{i},Y_{i}=\left[y_{i}\left(k\right)/y_{i}(1)|k=0,1,2\ldots ,n)\right]$$
  The dimensionalization of comparative sequences is
  (5) $$Y_{j},Y_{j}=\left[y_{j}\left(k\right)/y_{j}(1)|k=0,1,2\ldots ,n)\right]$$
(3) Calculate the grey correlation coefficient of each material performance index using the following formula:
  (6) $$\xi_{j}\left(k\right)=|\frac{\Delta_{min}+\rho \Delta_{max}}{\Delta (k)+\rho \Delta_{max}}|$$
  (7) $$\Delta_{min}=min|y_{i}\left(k\right)-y_{j}\left(k\right)|$$
  (8) $$\Delta_{max}=max|y_{i}\left(k\right)-y_{j}\left(k\right)|$$
  (9) $$\Delta (k)=|y_{i}\left(k\right)-y_{j}\left(k\right)|$$
  In the equation, ρ is the resolution coefficient, with a value range of (0, 1), which is general.
(4) Calculate the correlation degree of various material performance indicators using the following formula:
  (10) $$\gamma_{j}=\frac{1}{n}\sum_{k=1}^{n}\xi_{j}\left(k\right)$$

According to Equations (2) to (10), using the averaging method, with $E^{\prime }(t)$ as the reference sequence and adhesion force and the DMT modulus as the comparative sequence, the grey correlation method was used to analyze the correlation between the asphalt’s micro-mechanical indicators and the asphalt mixture’s macro-mechanical indicators. The correlation results are shown in Table 7.

According to Table 7, it was found that the grey correlation coefficient between the adhesion force index of the asphalt and the $E^{\prime }(t)$ index of the asphalt mixtures was 0.711, and the grey correlation coefficient between the DMT modulus and the $E^{\prime }(t)$ index was 0.767. This indicated a strong correlation between the micro-mechanical properties of the asphalt and the low-temperature cracking resistance of the asphalt mixtures, suggesting that changes in the micro-mechanical properties of asphalt have a significant impact on the low-temperature cracking resistance of the asphalt mixtures. Compared to the adhesion force index of the asphalt, the correlation between the DMT modulus of the asphalt and the $E^{\prime }(t)$ index of the asphalt mixtures was stronger, indicating that the deformation ability of asphalt has a greater influence on the low-temperature cracking resistance of asphalt mixtures.

## 5. Conclusions

This study investigated the low-temperature crack resistance of high-dosage crumb-rubber-modified asphalt and a high-dosage mixture under freeze–thaw cycles. The novelty of this study lies in the fact that it conducted freeze–thaw cycle tests on asphalt. The effects of the crumb rubber and warm-mixing additives on the asphalt and asphalt mixtures were explored, as well as the correlation between the asphalt micro-mechanics and asphalt mixture. The main research findings were as follows:

(i) Under the same number of freeze–thaw cycles, the salt solution caused more damage to the asphalt than water, making the asphalt harder and reducing its deformation ability, thus negatively affecting the low-temperature crack resistance of the asphalt mixtures.
(ii) Increasing the dosage of the rubber powder made the surface of the asphalt rougher, increasing the adhesive area and improving the adhesion between the asphalt and the aggregates, thereby enhancing the crack resistance of the asphalt mixtures. However, the increased dosage of the rubber powder also reduced the deformation ability of the asphalt.
(iii) Adding warm-mixing additives to the asphalt mixtures improved their low-temperature crack resistance, and SDYK had a better effect than EM in improving the low-temperature crack resistance of the asphalt mixtures, indicating that SCR-30WMA had the best low-temperature crack resistance.
(iv) There was a significant correlation between the micro-mechanical properties of the asphalt and the low-temperature crack resistance of the asphalt mixtures. Compared to the adhesion force index, DMT had a stronger correlation with E′(t), and the influence of the asphalt deformation ability on the low-temperature crack resistance of the asphalt mixtures was more significant.

## Figures and Tables

**Table 1:** Test materials and test conditions and their markings.

Types of Test Materials and Conditions	Marking
modified asphalt with 20% rubber powder content	CR-20
EM-modified asphalt with 20% rubber powder content	ECR-20
SDYK-modified asphalt with 20% rubber powder content	SCR-20
modified asphalt with 30% rubber powder content	CR-30
EM-modified asphalt with 30% rubber powder content	ECR-30
SDYK-modified asphalt with 30% rubber powder content	SCR-30
asphalt mixture with 20% rubber powder content	CR-20HMA
EM asphalt mixture with 20% rubber powder content	ECR-20WMA
SDYK asphalt mixture with 20% rubber powder content	SCR-20WMA
asphalt mixture with 30% rubber powder content	CR-30HMA
EM asphalt mixture with 30% rubber powder content	ECR-30WMA
SDYK asphalt mixture with 30% rubber powder content	SCR-30WMA
5, 10, 15, and 20 water freeze–thaw cycles	5-0, 10-0, 15-0, 20-0
5, 10, 15, 20 salt freeze–thaw cycles	5-8, 10-8, 15-8, 20-8

**Table 2:** Performance index for 60-mesh rubber particle.

Technical Index	Technical Requirement	Test Result
Volume density (g/cm^3^)	1.15 ± 0.05	0.89
Ash content (%)	≤8	6
Fe content (%)	≤0.03	0.021
Heating loss (%)	≤1	0.6
Fiber content (%)	<1	0.4

**Table 3:** Technical indicators of 20% rubber-powder-modified asphalt.

Technical Index	Technical Requirement	CR-20	ECR-20	SCR-20
Penetration at 25 °C, 100 g, 5 s, 0.1 mm (mm)	40~70	68.4	62.7	53.2
Softening point (°C)	≥60	58.3	60.5	64.4
Ductility at 5 °C (mm)	≥100	174	186	195
Viscosity at 175 °C (Pa.s)	2~4	2.6	2.1	2.4

**Table 4:** Technical indicators of 30% rubber-powder-modified asphalt.

Technical Index	Technical Requirement	CR-30	ECR-30	SCR-30
Penetration at 25 °C, 100 g, 5 s, 0.1 mm (mm)	40~70	50.9	50.2	49.2
Softening point (°C)	≥60	78.4	80.5	82.8
Ductility at 5 °C (mm)	≥100	201	215	223
Viscosity at 175 °C (Pa.s)	2~4	3.0	2.6	2.7

**Table 5:** Volume index under optimal asphalt dosage.

Aggregates	Bulk Density (g/cm^3^)	Theoretical Density (g/cm^3^)	VMA (%)	Porosity (%)	VFA (%)	Stability (KN)	Stream Value (0.1 mm)
CR-20HMA	2.457	2.554	15.8	4.5	71.6	8.23	3.7
CR-30HMA	2.454	2.567	16.4	4.4	73.1	10.03	3.4
Technical requirement	—	—	≥14.5	3–5	70–85	≥8	2–4

**Table 6:** Mix design results.

Types of Materials Used	10–20 mm	5–10 mm	0–5 mm	Mineral Powder	CR-20HMA Optimum Asphalt Content	CR-30HMA Optimum Asphalt Content
Dosage/%	29	30	38	3	5.8	6.2

**Table 7:** Correlation coefficients and correlations of asphalt and asphalt mixture’s mechanical property indexes.

Asphalt Type and Freeze–Thaw Cycling Effect	Evaluating Indicator	
	Adhesive Force	DMT Modulus
SCR-30WMA	1	1
ECR-30WMA	0.962987	0.941433
CR-30HMA	0.860265	0.84927
SCR-20WMA	0.762976	0.719885
ECR-20WMA	0.762254	0.766684
CR-20HMA	0.734582	0.75138
SCR-30WMA—20-0	0.423939	0.455544
ECR-30WMA—20-0	0.487058	0.530289
CR-30HMA—20-0	0.649492	0.708061
SCR-20WMA—20-0	0.742307	0.899695
ECR-20WMA—20-0	0.859614	0.928224
CR-20HMA—20-0	0.967183	0.980874
SCR-30WMA—20-8	0.333333	0.372169
ECR-30WMA—20-8	0.393766	0.450119
CR-30HMA—20-8	0.59645	0.70072
SCR-20WMA—20-8	0.642517	0.82563
ECR-20WMA—20-8	0.755187	0.9223
CR-20HMA—20-8	0.855239	0.996094
Grey relational grade	0.711	0.767

## Data Availability

Data are contained within the article.
